# Meaningful Aging in the Face of Vulnerability: Perspectives From the Humanities and Arts

**DOI:** 10.1093/geroni/igaa057.3068

**Published:** 2020-12-16

**Authors:** Kate de Medeiros, Desmond O’Neill

## Abstract

This symposium interprets GSA’s 2020 leading conference theme, “Why Age Matters”, as touching upon fundamental existential questions about the meaning of old age. Although meanings of aging have always been implicitly present in a variety of disciplinary gerontological studies, scholars from the humanities and arts have traditionally taken the lead in the field to provide thorough reflections and analyses about what makes later life meaningful. In this symposium, we aim to present a selection of perspectives from the humanities and arts that explore how meaningful aging can be realized in circumstances of the increasing vulnerability that inevitably accompanies old age. First, Hanne Laceulle uses a practice-theoretical philosophical framework to argue that the common assumption that vulnerability constitutes a threat to meaningfulness deserves to be nuanced, because meaning can also occur in the process of integrating vulnerability in one’s life. Second, Theresa Allison, Jennie Gubner and Alexander Smith show how vulnerable older adults living with dementia and their caregivers seek meaning in daily life, adapting meaningful activities to circumstances of increasing vulnerability. Third, Kate de Medeiros and Ulla Kriebernegg discuss how a dialogue between facts and fictions, narrative and literary gerontology, can contribute in seeing vulnerability as a form of resistance. Fourth, Margaret Perkinson illustrates the power of visual images as elicitors of reflections on meaning among the older inhabitants of a Guatemalan village. Documenting villagers’ own perspectives through PhotoVoice methodology underscores the fundamental importance of taking first-person perspectives into account when studying meaning.

